# Lower serum sodium level predicts higher risk of infection-related hospitalization in maintenance hemodialysis patients: an observational cohort study

**DOI:** 10.1186/1471-2369-14-276

**Published:** 2013-12-19

**Authors:** Shintaro Mandai, Michio Kuwahara, Yuri Kasagi, Keita Kusaka, Tomomi Tanaka, Satomi Shikuma, Wataru Akita, Sei Sasaki

**Affiliations:** 1 Department of Nephrology, Shuuwa General Hospital, 1200 Yaharashinden, Kasukabe, Saitama 344-0035, Japan; 2 Department of Nephrology, Graduate School of Medicine, Tokyo Medical and Dental University, 1-5-45 Yushima, Bunkyo-ku, Tokyo 113-8519, Japan

**Keywords:** End-stage renal disease, Hemodialysis, Hyponatremia, Infection, Mortality

## Abstract

**Background:**

Hyponatremia is associated with increased mortality in chronic kidney disease with and without end-stage renal disease (ESRD). Increasing evidence suggests that hyponatremia is not only a marker of severe underlying disease, but also a direct contributor to mortality. However, specific pathogenesis or diseases contributing to mortality in the hyponatremic population are unknown. This study aimed to clarify the relationship between serum sodium level (sNa) and infection risk in ESRD patients.

**Methods:**

This observational cohort study included 332 patients on maintenance hemodialysis in our dialysis unit in May 2009. The mean of 3 monthly measurements of glucose-corrected sNa before each dialysis session in May, June, and July 2009 was applied as baseline sNa. The primary endpoint was first infection-related hospitalization (IRH), and the secondary endpoint was death of any cause. Data were analyzed using Cox hazards modeling, adjusted for baseline demographics and characteristics, or laboratory data. Patients were followed until transfer, kidney transplantation, death, or study end on January 31, 2013.

**Results:**

Mean sNa was 138.9 mEq/L (1st tertile: <138.0, n = 104; 2nd tertile: 138.0–140.0, n = 116; 3rd tertile: >140.0, n = 112). During 39.5 months’ mean follow-up, 57 patients experienced IRH (56.4/1,000 patient-years overall; 89.7/1,000 in 1st tertile; 57.9/1,000 in 2nd tertile; 28.0/1,000 in 3rd tertile), and 68 patients died. The hazard ratio (HR) for IRH was higher for the 1st and 2nd tertiles than the 3rd tertile (unadjusted HR, 3.20; 95% confidence interval (CI), 1.54–6.64; p = 0.002; adjusted HR, 2.36; 95% CI, 1.10–5.04; p = 0.027; and unadjusted HR, 2.07; 95% CI, 0.98–4.40; p = 0.058; adjusted HR, 2.11; 95% CI, 0.99–4.51; p = 0.054 respectively). In a continuous model, higher sNa was associated with lower risk of IRH (adjusted HR, 0.90; 95% CI, 0.81–0.99; p = 0.040), and lower all-cause mortality (adjusted HR, 0.91; 95% CI, 0.83–1.00; p = 0.049).

**Conclusions:**

Lower sNa is an independent predictor of higher risk for infection-related hospitalization in maintenance hemodialysis patients. Infectious disease may partially account for the increased mortality observed in the hyponatremic population with ESRD.

## Background

Hyponatremia is among the most common electrolyte disorders in clinical practice, particularly in hospitalized patients, 15% to 40% of whom have hyponatremia, depending on the definition [1,2]. The relationship between hyponatremia and increased mortality has been recognized, primarily in patients with heart failure and liver cirrhosis [3-7]. Recent studies have additionally revealed this association in a wide variety of diseases including myocardial infarction [2,8], pulmonary embolism [9], cancer [2,10] and pneumonia [11], and in patients in perioperative settings [1,12] and those in intensive care units [13]. Similar observations have been made in chronic kidney disease (CKD) patients with [14-16] and without end-stage renal disease (ESRD) [17]. These studies have identified hyponatremia as an independent predictor of mortality after adjustments for potential confounders reflecting severity of underlying disease, and thus suggested that hyponatremia is not only a marker of severe underlying disease, but also a direct contributor to mortality. However, it has yet to be clarified how hyponatremia contributes to mortality, or which disease is responsible for increased mortality in the hyponatremic population. Previous data which answer this question are very limited, but Waikar et al. [14] showed that the relationship between lower sNa and higher mortality was not significant in analyses of cardiovascular mortality, but rather, was significant in analyses of non-cardiovascular mortality in maintenance hemodialysis (HD) patients. We hypothesized that hyponatremia is associated with increased susceptibility to infection, and infectious disease additionally contribute to mortality in the hyponatremic population. This study aimed to investigate the relationship between sNa and the future risk of hospitalization due to infectious disease in patients with ESRD.

## Methods

### Study population

In May 2009, 381 patients underwent maintenance HD in our dialysis unit. The study participants included 354 patients whose HD vintage was 12 months or more. Participants were excluded if patients lacked at least one measurement among the 3 monthly routine laboratory measurements before each dialysis session in May, June, and July 2009 (n = 12), or an observational period was less than 6 months (n = 10). The remaining 332 patients were enrolled in this study. All patients received thrice-weekly HD. Cardiovascular disease (CVD) was defined as myocardial infarction, coronary revascularization, or hospitalization for heart failure. Peripheral vascular disease was defined as revascularization of arteries distal to the common iliac artery or amputation. Cerebrovascular disease was defined as cerebral infarction, cerebral hemorrhage, or subarachnoid hemorrhage. Chronic liver disease was defined as liver cirrhosis or chronic viral hepatitis. Dialysis vintage was defined as time from initiation of HD until May 1, 2009, or from that of peritoneal dialysis if patients had undergone peritoneal dialysis before switching to HD. Intradialytic weight loss (IDWL) (% per body weight) was calculated according to an equation: IDWL = intradialytic filtration volume in kg/post-dialytic body weight in kg × 100. In our dialysis unit, regular blood sampling was conducted monthly before the first dialysis session in a week (on Mon or Tue). The sNa was measured in our hospital’s clinical laboratory using an electrode-sensitive assay (CA-25011, JEOL Ltd., Japan), and precision measurement and calibration are routinely conducted every day. Although data in 2009 were not available, the latest data of 30 measurements during September 2013 were available: the mean value of the lower-concentration standard solution widely used in Japan (mean value of the previous cumulative measurements using the same method at multiple centers in Japan, 132.37 mEq/L) was 131.70 ± 0.64 mEq/L (95% confidence interval, 131.46–131.94), and that of the higher-concentration standard solution (mean value of the previous cumulative measurements at multiple centers in Japan, 150.05 mEq/L) was 149.28 ± 0.49 mEq/L (95% confidence interval, 149.10–149.46). The sNa was adjusted with the paired serum glucose level if the serum glucose level was over 200 mg/dL (1.6 mEq/L per 100 mg/dL) [18]. The mean of the 3 monthly measurements of body mass index (BMI), IDWL, and laboratory data was applied as each baseline value, with the exception of β2-microglobulin (β2-MG), which was measured routinely once in 3 months in all participants. Patients were followed from May 1, 2009 until transfer, kidney transplant, death, or study end on January 31, 2013.

This study protocol was approved by the ethics committee of Shuuwa General Hospital, and the study was preformed in accordance with the Declaration of Helsinki guidelines regarding ethical principles for medical research involving human subjects. Informed consent was obtained by all participants after information about the study was displayed in poster format in a public space.

### Outcomes

The primary outcome was time to first infection-related hospitalization (IRH) after the 3rd laboratory measurement in July 2009. Events after the second event were not included in the analysis. IRH was defined when primary diagnosis on admission was infectious disease as noted in medical records. IRHs were divided into seven exclusive categories: pulmonary infections (e.g., pneumonia, bronchopneumonia), gastrointestinal infections (e.g., appendicitis, enterocolitis, cholecystitis, cholangitis), genitourinary infections (e.g., urinary tract infection, prostatitis), soft tissue infections (e.g., cellulitis), joint or bone infections (e.g., septic arthritis, osteomyelitis), septicemia, and others. Catheter-related bloodstream infection and graft infection were categorized as septicemia. The secondary outcome was death of any cause.

### Statistical analysis

Data are described as means and standard deviations for continuous variables, or numbers and percentages for categorical variables. The association of baseline demographics and characteristics, or laboratory data with sNa, was examined using univariate and multivariate linear regression analyses. In multivariate analyses, covariates included those that were statistically correlated with baseline sNa with p < 0.05 at univariate analyses. Kaplan-Meier curves were used for analysis of time to first IRH or death of any cause. Predictors of outcomes were evaluated using Cox hazards modeling adjusted for sNa, all covariates, which were correlated to the outcomes with p < 0.001 at univariate analyses, and also the determinants of baseline sNa at multivariate linear regression analyses.

Statistical analyses were performed with Dr. SPSS II for Windows, a software package based on SPSS 11.0 J for Windows (SPSS, Chicago, IL, USA). P*-*values < 0.05 were considered significant for all analyses.

## Results

### General characteristics of participants

In total, 332 HD patients were enrolled into this study. The baseline demographics and characteristics are shown in Table 1. The mean age of the study subjects was 61.6 ± 12.6 years, 36% of the study subjects were female, 36% were current or past smokers, 8% were not able to ambulate or transfer, 36% had diabetes mellitus (DM), and 30% had CVD. The study participants were *categorized* into three groups based on sNa; 1st tertile: < 138.0 mEq/L, n = 104; 2nd tertile: 138.0–140.0 mEq/L, n = 116; 3rd tertile: > 140.0 mEq/L, n = 112.

**Table 1:** Baseline demographics and characteristics of the study participants

		**Serum sodium level tertile (mEq/L)**		
	**Whole group**	**1st tertile <138.0**	**2nd tertile 138.0 – 140.0**	**3rd tertile >140.0**
Number of patients, n (%)	332 (100)	104 (31)	116 (35)	112 (34)
Age (years)	61.6 ± 12.6	62.3 ± 14.2	60.6 ± 12.0	61.9 ± 11.5
Female, n (%)	121 (36)	34 (33)	41 (35)	46 (41)
Body mass index (kg/m^2^)	21.2 ± 3.6	20.9 ± 4.1	21.2 ± 3.4	21.5 ± 3.3
Smoking history, n (%)	118 (36)	38 (37)	39 (34)	41 (37)
Inability to ambulate or transfer, n (%)	28 (8)	11 (11)	9 (8)	8 (7)
Comorbid conditions				
Diabetes mellitus, n (%)	120 (36)	51 (49)	36 (31)	33 (29)
Hypertension, n (%)	310 (93)	100 (96)	105 (91)	105 (94)
Cardiovascular disease, n (%)	101 (30)	42 (40)	30 (26)	29 (26)
Peripheral vascular disease, n (%)	16 (5)	9 (9)	4 (3)	3 (3)
Cerebrovascular disease, n (%)	47 (14)	19 (18)	13 (11)	15 (13)
Chronic liver disease, n (%)	37 (11)	11 (11)	13 (11)	13 (12)
Cancer, n (%)	43 (13)	7 (7)	16 (14)	20 (18)
Dialysis vintage (months)	124 ± 96	102 ± 79	135 ± 94	134 ± 108
IDWL (% of body weight)	5.1 ± 1.3	5.6 ± 1.4	5.0 ± 1.3	4.8 ± 1.2
Vascular access				
Fistula	290 (87)	88 (85)	103 (89)	99 (88)
Graft	38 (12)	16 (15)	12 (10)	10 (9)
Catheter	4 (1)	0 (0)	1 (1)	3 (3)
Laboratory data				
Serum sodium (mEg/L)	138.9 ± 2.6	136.0 ± 1.8	139.0 ± 0.7	141.5 ± 1.1
Hemoglobin (g/dL)	10.5 ± 0.9	10.7 ± 1.1	10.5 ± 0.9	10.4 ± 0.8
Serum albumin (g/dL)	3.8 ± 0.3	3.8 ± 0.3	3.9 ± 0.3	3.8 ± 0.3
Serum creatinine (mg/dL)	11.7 ± 2.8	11.4 ± 3.0	12.1 ± 2.8	11.7 ± 2.7
Serum β2-microglobulin (mg/dL)	27.3 ± 5.3	28.0 ± 4.9	27.2 ± 5.8	26.6 ± 5.1

Values are expressed as mean ± SD for continuous variables, or numbers and percentages for categorical variables.

*Abbreviations*: *IDWL* intradialytic weight loss.

### Determinants of baseline serum sodium level

In univariate linear regression analyses, DM, CVD, cancer, dialysis vintage, IDWL, hemoglobin, and serum albumin were correlated with baseline sNa (Table 2). In multivariate analyses, cancer and serum albumin were positively correlated, and IDWL and hemoglobin were negatively correlated with sNa (Table 2).

**Table 2:** Determinants of baseline serum sodium level

	**Univariate**		**Multivariate**^**a**^	
	**Coefficient**	**p-value**	**Coefficient**	**p-value**
Age (per 5 years)	-0.008	0.886		
Female (vs male)	0.426	0.147		
Body mass index (per 1 kg/m^2^)	0.004	0.910		
Smoking history	-0.078	0.793		
Inability to ambulate or transfer	-0.577	0.257		
Comorbid conditions				
Diabetes mellitus	-1.006	0.001	-0.507	0.107
Hypertension	-0.505	0.374		
Cardiovascular disease	-0.818	0.008	-0.409	0.170
Peripheral vascular disease	-1.139	0.084		
Cerebrovascular disease	-0.158	0.698		
Chronic liver disease	-0.395	0.380		
Cancer	1.185	0.005	1.246	0.002
Dialysis vintage (per 12 months)	0.045	0.012	0.023	0.220
IDWL (per 1% of body weight)	-0.554	<0.001	-0.479	<0.001
Vascular access				
Fistula	0 (ref)			
Graft or catheter	-0.408	0.338		
Laboratory data				
Hemoglobin (per 1 g/dL)	-0.357	0.018	-0.428	0.003
Serum albumin (per 1 g/dL)	1.122	0.019	1.349	0.003
Serum creatinine (per 1 mg/dL)	0.041	0.414		
Serum β2-microglobulin (per 5 mg/dL)	-0.249	0.061		

^a^Adjustments in all covariates, which were significantly correlated with baseline serum sodium level, with p < 0.05 at univariate analyses including diabetes mellitus, cardiovascular disease, cancer, dialysis vintage, IDWL, hemoglobin, and serum albumin.

*Abbreviations*: *IDWL* intradialytic weight loss.

### Primary outcome

**Figure 1:**
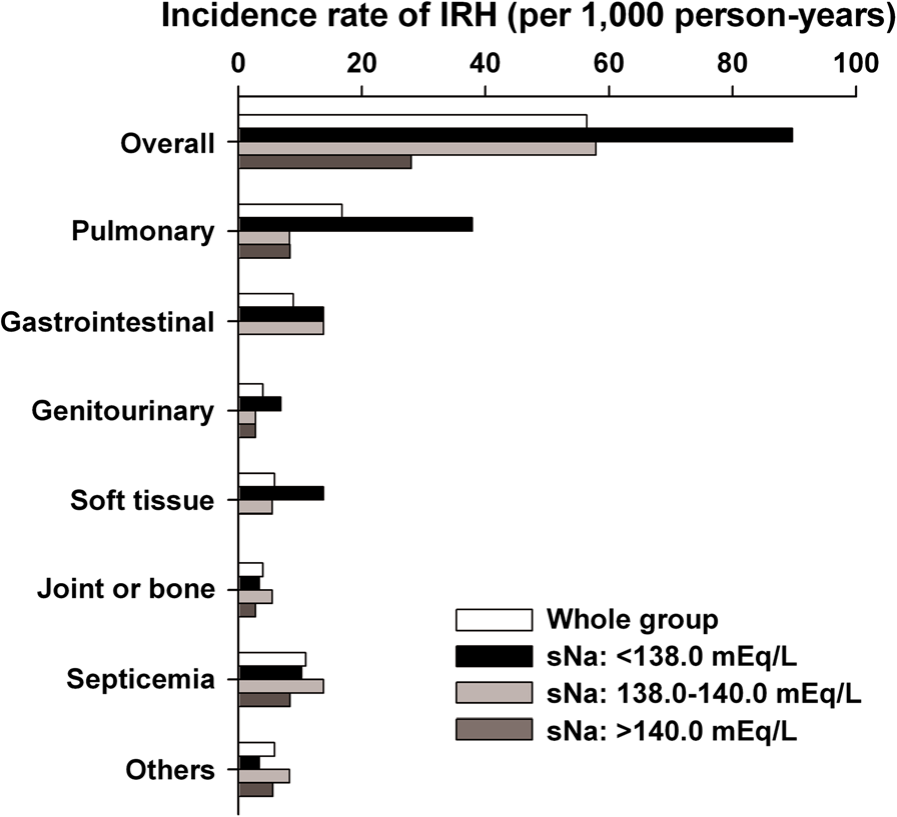
**Incidence rate of each subtype of infection-related hospitalization according to serum sodium level tertiles.** Abbreviations: IRH, infection-related hospitalization; sNa, serum sodium level.

During a mean follow-up of 39.5 months, 57 patients experienced at least one IRH (incidence rate: 56.4/1,000 patient-years overall; 89.7/1,000 in sNa 1st tertile; 57.9/1,000 in 2nd tertile; 28.0/1,000 in 3rd tertile). The incidence rate of each IRH subtype is shown in Figure 1. Pulmonary infections were the most common type of infection among the HD patients in this study. The types of IRH having the highest incidence in the 1st tertile were observed to be pulmonary infections, gastrointestinal infections, genitourinary infections, and soft tissue infections.

**Figure 2:**
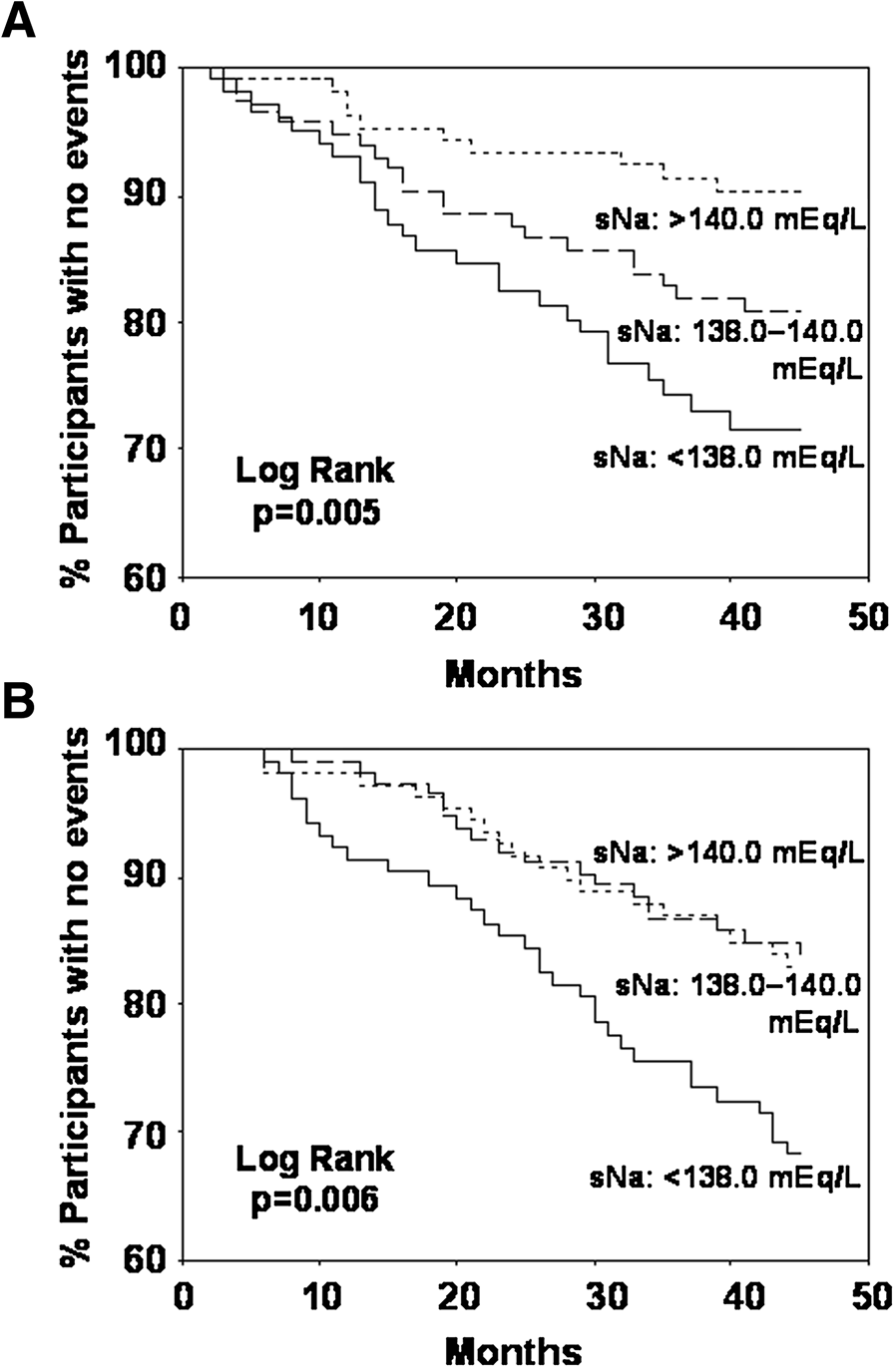
**Kaplan-Meier curves of time to outcomes according to serum sodium level tertiles. (A)** First infection-related hospitalization. **(B)** Death of any cause. Abbreviations: sNa, serum sodium level.

The lower sNa tertiles were significantly associated with higher risk of IRH (Figure 2A). More IRH events occurred in the 1st and 2nd tertiles than in the 3rd tertile at univariate and multivariate Cox models (Table 3). When sNa was considered as a continuous variable, lower sNa was associated with higher risk of IRH (Table 3). CVD, and graft or catheter were also associated with increased risk of IRH.

**Table 3:** Predictors of infection-related hospitalization in the study population

	**Univariate**		**Multivariate**^**a**^	
	**HR (95% CI)**	**p-value**	**HR (95% CI)**	**p-value**
Serum sodium level				
Categorical model				
<138.0 mEq/L	3.20 (1.54–6.64)	0.002	2.36 (1.10–5.04)	0.027
138.0 – 140.0 mEq/L	2.07 (0.98–4.40)	0.058	2.11 (0.99–4.51)	0.054
>140.0 mEq/L	1 (ref)		1 (ref)	
Continuous model (per 1 mEq/L)	0.86 (0.79–0.94)	<0.001	0.90 (0.81–0.99)	0.040
Age (per 5 years)	1.06 (0.95–1.18)	0.318		
Female (vs male)	1.31 (0.78–2.22)	0.313		
Body mass index (per 1 kg/m^2^)	0.99 (0.91–1.07)	0.711		
Smoking history	1.52 (0.90–2.56)	0.120		
Inability to ambulate or transfer	4.79 (2.61–8.80)	<0.001	2.10 (0.90–4.91)	0.086
Comorbid conditions				
Diabetes mellitus	1.66 (0.99–2.80)	0.056		
Hypertension	0.65 (0.28–1.51)	0.313		
Cardiovascular disease	2.54 (1.51–4.27)	<0.001	2.21 (1.30–3.75)	0.003
Peripheral vascular disease	1.83 (0.66–5.07)	0.243		
Cerebrovascular disease	1.65 (0.86–3.19)	0.135		
Chronic liver disease	1.08 (0.49–2.39)	0.846		
Cancer	1.01 (0.46–2.23)	0.976		
Dialysis vintage (per 12 months)	0.98 (0.95–1.01)	0.242		
IDWL (per 1% of body weight)	1.05 (0.86–1.28)	0.651		
Vascular access				
Fistula	1 (ref)		1 (ref)	
Graft or catheter	4.08 (2.33–7.15)	<0.001	2.95 (1.57–5.55)	0.001
Laboratory data				
Hemoglobin (per 1 g/dL)	0.80 (0.59–1.08)	0.140		
Serum albumin (per 1 g/dL)	0.16 (0.07–0.37)	<0.001	0.47 (0.15–1.45)	0.186
Serum creatinine (per 1 mg/dL)	0.84 (0.76–0.92)	<0.001	0.97 (0.86–1.10)	0.654
Serum β2-microglobulin (per 5 mg/dL)	1.26 (1.01–1.57)	0.037		

^a^Adjustments in serum sodium level, all covariates, which were correlated with the outcome, with p < 0.001 at univariate analyses including inability to ambulate or transfer, cardiovascular disease, graft or catheter, serum albumin, and serum creatinine, and also determinants of baseline serum sodium level at multivariate linear regression analyses including cancer, IDWL, and hemoglobin.

*Abbreviations*: *CI* confidence interval, *HR* hazard ratio, *IDWL* intradialytic weight loss.

### Secondary outcome

During follow-up, 68 patients died. The association of sNa tertile with all-cause mortality was statistically significant (Figure 2B). When sNa was considered as a continuous variable, the relationship between lower sNa and higher all-cause mortality was observed in Cox models (Table 4). Older age, DM, and cancer were also associated with increased all-cause mortality. The association of CVD with increased all-cause mortality was marginally significant.

**Table 4:** Predictors of all-cause mortality in the study population

	**Univariate**		**Multivariate**^**a**^	
	**HR (95% CI)**	**p-value**	**HR (95% CI)**	**p-value**
Serum sodium level				
Categorical model				
<138.0 mEq/L	2.06 (1.15–3.66)	0.014	1.57 (0.85–2.87)	0.148
138.0 – 140.0 mEq/L	0.94 (0.49–1.81)	0.853	0.97 (0.50–1.89)	0.938
>140.0 mEq/L	1 (ref)		1 (ref)	
Continuous model (per 1 mEq/L)	0.87 (0.81–0.95)	0.001	0.91 (0.83–1.00)	0.049
Age (per 5 years)	1.40 (1.25–1.57)	<0.001	1.24 (1.08–1.41)	0.002
Female (vs male)	0.76 (0.46–1.28)	0.302		
Body mass index (per 1 kg/m^2^)	0.88 (0.81–0.95)	0.001		
Smoking history	1.32 (0.81–2.13)	0.267		
Inability to ambulate or transfer	4.13 (2.32–7.36)	<0.001	1.44 (0.69–2.99)	0.328
Comorbid conditions				
Diabetes mellitus	2.80 (1.73–4.54)	<0.001	1.98 (1.17–3.36)	0.011
Hypertension	1.66 (0.52–5.30)	0.388		
Cardiovascular disease	2.36 (1.47–3.80)	<0.001	1.65 (0.98–2.76)	0.059
Peripheral vascular disease	3.84 (1.90–7.75)	<0.001	1.89 (0.88–4.06)	0.104
Cerebrovascular disease	2.07 (1.18–3.63)	0.011		
Chronic liver disease	1.63 (0.85–3.10)	0.140		
Cancer	2.82 (1.65–4.84)	<0.001	2.49 (1.39–4.46)	0.002
Dialysis vintage (per 12 months)	0.99 (0.95–1.02)	0.357		
IDWL (per 1% of body weight)	1.02 (0.85–1.23)	0.824		
Vascular access				
Fistula	1 (ref)			
Graft or catheter	2.14 (1.20–3.79)	0.010		
Laboratory data				
Hemoglobin (per 1 g/dL)	0.82 (0.62–1.07)	0.140		
Serum albumin (per 1 g/dL)	0.16 (0.08–0.32)	<0.001	0.65 (0.24–1.74)	0.388
Serum creatinine (per 1 mg/dL)	0.76 (0.69–0.83)	<0.001	0.93 (0.81–1.06)	0.272
Serum β2-microglobulin (per 5 mg/dL)	1.26 (1.03–1.54)	0.027		

^a^Adjustments in serum sodium level, all covariates, which were correlated with the outcome, with p < 0.001 at univariate analyses including age, inability to ambulate or transfer, diabetes mellitus, cardiovascular disease, peripheral vascular disease, cancer, serum albumin, and serum creatinine, and also determinants of baseline serum sodium level at multivariate linear regression analyses including IDWL and hemoglobin.

*Abbreviations*: *CI* confidence interval, *HR* hazard ratio, *IDWL* intradialytic weight loss.

## Discussion

The principal finding in the current study is that lower sNa is an independent predictor of higher risk for infection-related hospitalization in maintenance HD patients. To the best of our knowledge, this study is the first to investigate the relationship between sNa and risk of infection in patients with ESRD. Although increasing evidence indicates a possible causal relationship between sNa and mortality, specific pathogenesis or diseases contributing to mortality in the hyponatremic population have yet to be clarified. Infectious disease may partially account for the increased mortality observed in hyponatremic subjects with ESRD.

The present study showed that lower sNa is an independent predictor of higher risk for infection in ESRD patients, and confirmed the established relationship between lower sNa and higher mortality, even after adjustments for various covariates. Prior studies have shown an association between hyponatremia and increased mortality in a wide variety of diseases [3-11]. However, in the majority of these studies, baseline sNa was based on measurements during hospitalization and/or in the acute phase of underlying disease, and these settings are more likely to increase either an appropriate or inappropriate secretion of arginine vasopressin (antidiuretic hormone, ADH), resulting in higher reabsorption of solute-free water with a lower level of sNa. In contrast, recent large cohort studies in the general population [19,20] or in CKD patients [17] have shown that chronic and mild hyponatremia are associated with increased all-cause mortality, even after adjustments for potential confounders or underlying disease. Furthermore, Waikar et al. [14] showed a continuous relationship between lower sNa and higher mortality in the HD population without residual renal function, indicating that this relationship is independent of ADH secretion related to underlying disease. Thus, it is possible that hyponatremia is not only a marker of severe underlying disease that results in poor outcome, but is also a direct contributor to mortality [21]. Similarly, our findings from the HD population suggest that hyponatremia possibly results in increased susceptibility to infection, and infectious disease additionally contributes increased mortality in hyponatremic subjects with ESRD. Although several studies of the HD population have not clarified specific diseases that are responsible for increased mortality in hyponatremic subjects [14-16], the HEMO study showed that the relationship between lower sNa and higher mortality was not significant in analyses of cardiovascular mortality, but rather, was significant in analyses of non-cardiovascular mortality [14]. The findings of the present study may explain these observations.

Hyponatremia is a relatively frequent complication observed in infectious disease, especially in tuberculosis [22], pneumonia [11], human immunodeficiency virus infection [23], and bacterial meningitis [24], and the association of hyponatremia with increased mortality in these diseases has been identified. It is well known that infection can result in hyponatremia through various pathopysiological mechanisms including hypovolemia, hyperglycemia, renal failure, heart failure, or syndrome of inappropriate ADH secretion, but it has been unknown whether the existence of hyponatremia predicts infection. Of note is that the findings of the present study showed that subjects with lower sNa are more likely to develop infectious disease, although the study population was specific. Previously, a number of studies examined risk factors for IRH or infectious death in ESRD patients, but no studies considered sNa as a covariate related to risk for infection. However, the present study included almost all covariates that are known risk factors including age [25-28], DM [25-27], CVD [26], inability to ambulate or transfer [26], vascular access [25], hemoglobin [26], serum albumin [25-28], and serum β2-MG [28]. A few studies in hospitalized patients have shown that hyponatremia can be a risk of infection, but the data are very limited. In an analysis of perioperative patients, preoperative hyponatremia predicted wound infections and pneumonia [12]. Jensen et al. showed that hyponatremia was one of the risks factors for hospital-acquired *Staphylococcus aureus* bacteremia in hospitalized patients [29]. There is a need for further studies to investigate the relationship between sNa and risk of infection also in the general population.

A key question is whether hyponatremia is merely a marker of severe comorbidities, or is a direct contributor to mortality; similarly, it remains unknown whether hyponatremia is merely an indicator of decreased immunological defense related to underlying disease, or results in increased susceptibility to infection. The present observational study could not answer this question. Given that data on urine output for each participant in the present study were not available, patients whose HD vintage was less than 12 months were excluded to decrease the influence of residual renal function with ADH secretion related to underlying disease on sNa. Nonetheless, residual confounders may have remained. An interventional study controlling for sNa is particularly difficult in ESRD patients, given that a vasopressin V2-receptor antagonist is not effective in oligoanuric subjects, or that a change in the dialysate sodium level does not influence pre-dialytic sNa [16]. There are several possible explanations for the pathophysiological link between sNa and susceptibility to infection. First, cellular edema of mucosal membrane associated with extracellular hypotonicity and water transfer into the intracellular space may result in decreased microbial barrier function of the mucosa in the respiratory, gastrointestinal, or urinary tract, given that infections of these organs were more frequent in the lower sNa tertile group in the present study (Figure 1). Second, decreased function of T_H_17 cells (interleukin-17 (IL-17)-producing helper T cells) may be related. T_H_17 cells and IL-17 play an important role in host immunity, with the capacity of protection against extracellular pathogens and fungi, whereas they are also important drivers of autoimmune disease, and have inflammatory properties. A recent study showed that elevated sodium chloride concentration promoted the differentiation of CD4^+^ T cells into T_H_17 cells *in vitro*, and a high-salt diet accelerated the development of autoimmune disease through the activation of T_H_17 cells in a mouse model [30]. Similarly, it is possible that elevated sodium chloride concentration increases protective function against microorganisms, whereas decreased sodium chloride concentration or hyponatremia can reduce it through inhibition of T_H_17 cells. Further studies are essential to examine the association of sNa with immunological parameters such as immunoglobulin levels, cytokine levels, or functions of immunocompetent cells including T_H_17 cells in order to clarify whether hyponatremia affects the immune system.

The current study had several limitations. First, this study was an observational, single-center study. Second, the sample size and the number of outcomes were limited, and may not be sufficient for generalization to a larger population. However, the mean sNa was almost comparable to that of the Japanese population according to the Dialysis Outcomes and Practice Patterns Study (DOPPS) [15], and our study population was representative of the Japanese HD population. The 1st tertile threshold value of sNa in this study was different from that in the DOPPS study [15], given the difference in mean sNa of the whole group. Similarly, the number of patients in the present study representing each subtype of IRH was limited, and it was not clear if the association of sNa with risk of IRH was independent of or specific for infection subtype (Figure 1). A larger cohort is needed to answer this question. Third, the relationship between sNa and risk of IRH in a long follow-up period was examined using only baseline sNa measurement. More than 3 monthly measurements of sNa were available during 6 months before IRH in all subjects who experienced IRH (n = 57), and the mean value of the 3 measurements available just before IRH was 137.4 ± 2.8 mEq/L. Although this value was significantly lower than the baseline value (137.9 ± 2.4 mEq/L) in the paired *t*-test, the difference was 0.5 mEq/L. Furthermore, 21 patients out of 26 whose baseline sNa was <138.0 mEq/L (1st sNa tertile) still had sNa <138.0 mEq/L just before IRH. Thus, the relationship between baseline sNa and risk of IRH would remain during a relatively long follow-up period. Fourth, data on dialysate sodium level for each participant were not available in the present study, and therefore were not included in the covariates. However, dialysate sodium level does not appear to determine pre-dialytic sNa [16], and it is controversial whether dialysate sodium level according to sNa is associated with mortality [15]. Finally, data on volume status that can influence sNa were limited. One to three monthly post-dialytic measurements of plasma brain natriuretic peptide were made from May to July 2009 in 257 of the 332 study patients. The mean values were 547 ± 965, 426 ± 667, and 370 ± 401 pg/mL in the 1st, 2nd, and 3rd tertiles, respectively. The differences among the three groups were not statistically significant, as assessed by the Kruskal-Wallis H test (p = 0.446).

## Conclusions

Lower sNa is an independent predictor of higher risk for infection-related hospitalization in maintenance HD patients. Infectious disease may partially account for the increased mortality observed in the hyponatremic population with ESRD.

## Abbreviations

ADH: Antidiuretic hormone; BMI: Body mass index; β2-MG: β2-microglobulin; CI: Confidence interval; CKD: Chronic kidney disease; CVD: Cardiovascular disease; DM: Diabetes mellitus; DOPPS: The dialysis outcomes and practice patterns study; ESRD: End-stage renal disease; HD: Hemodialysis; HR: Hazard ratio; IDWL: Intradialytic weight loss; IL-17: Interleukin-17; IRH: Infection-related hospitalization; sNa: Serum sodium level; TH17 cells: Interleukin-17-producing helper T cells.

## Competing interests

The authors declare that they have no competing interests.

## Authors’ contributions

SM performed the study design and statistical analysis, and drafted the manuscript. MK participated in the study design and coordination, and critically revised the manuscript. YK, KK, TT, SS, and WA contributed to treatment of the patients and collection of data. SS provided expert opinion, and critically revised the manuscript. All authors read and approved the final manuscript.

## Pre-publication history

The pre-publication history for this paper can be accessed here:

http://www.biomedcentral.com/1471-2369/14/276/prepub
